# Development of nanoparticles incorporated with quercetin and ACE2-membrane as a novel therapy for COVID-19

**DOI:** 10.1186/s12951-024-02435-2

**Published:** 2024-04-12

**Authors:** Jia-You Fang, Kuo-Yen Huang, Tong-Hong Wang, Zih-Chan Lin, Chin-Chuan Chen, Sui-Yuan Chang, En-Li Chen, Tai-Ling Chao, Shuenn-Chen Yang, Pan-Chyr Yang, Chi-Yuan Chen

**Affiliations:** 1 Research Center for Food and Cosmetic Safety and Research Center for Chinese Herbal Medicine, Chang Gung University of Science and Technology, Taoyuan, Taiwan; 2 Graduate Institute of Natural Products, Chang Gung University, Taoyuan, Taiwan; 3 Department of Anesthesiology, Chang Gung Memorial Hospital, Taoyuan, Taiwan; 4 Department of Clinical Laboratory Sciences and Medical Biotechnology, College of Medicine, National Taiwan University, Taipei, Taiwan; 5 National Taiwan University YongLin Institute of Health, Taipei, Taiwan; 6 Graduate Institute of Health Industry Technology, Research Center for Food and Cosmetic Safety and Research Center for Chinese Herbal Medicine, Chang Gung University of Science and Technology, Taoyuan, Taiwan; 7 Biobank, Chang Gung Memorial Hospital, Taoyuan, Taiwan; 8 Liver Research Center, Department of Hepato-Gastroenterology, Chang Gung Memorial Hospital, Taoyuan, Taiwan; 9 Chronic Diseases and Health Promotion Research Center, Chang Gung University of Science and Technology, Chiayi, Taiwan; 10 Department of Laboratory Medicine, National Taiwan University Hospital, Taipei, Taiwan; 11 Institute of Biomedical Sciences, Academia Sinica, Taipei, Taiwan; 12 Department of Internal Medicine, National Taiwan University Hospital and National Taiwan University College of Medicine, Taipei, Taiwan; 13 Genomics Research Center, Academia Sinica, Taipei, Taiwan; 14 No.1, Sec 1, Jen-Ai Rd, R.O.C, 100225 Taipei, Taiwan; 15 No.261, Wenhua 1st Rd., Guishan Dist, 33303 Taoyuan City, Taiwan; 16 Graduate School of Advanced Technology (Program for Precision Health and Intelligent Medicine), National Taiwan University, Taipei, Taiwan

**Keywords:** Nanoparticles, Quercetin, AXL, ACE2, COVID-19

## Abstract

**Introduction:**

Angiotensin-converting enzyme 2 (ACE2) and AXL tyrosine kinase receptor are known to be involved in the SARS-CoV-2 entry of the host cell. Therefore, targeting ACE2 and AXL should be an effective strategy to inhibit virus entry into cells. However, developing agents that can simultaneously target ACE2 and AXL remains a formidable task. The natural compound quercetin has been shown to inhibit AXL expression.

**Materials and methods:**

In this study, we employed PLGA nanoparticles to prepare nanoparticles encapsulated with quercetin, coated with ACE2-containing cell membranes, or encapsulated with quercetin and then coated with ACE-2-containing cell membranes. These nanoparticles were tested for their abilities to neutralize or inhibit viral infection.

**Results:**

Our data showed that nanoparticles encapsulated with quercetin and then coated with ACE2-containing cell membrane inhibited the expression of AXL without causing cytotoxic activity. Nanoparticles incorporated with both quercetin and ACE2-containing cell membrane were found to be able to neutralize pseudo virus infection and were more effective than free quercetin and nanoparticles encapsulated with quercetin at inhibition of pseudo virus and SARS-CoV-2 infection.

**Conclusions:**

We have shown that the biomimetic nanoparticles incorporated with both ACE-2 membrane and quercetin showed the most antiviral activity and may be further explored for clinical application.

**Supplementary Information:**

The online version contains supplementary material available at 10.1186/s12951-024-02435-2.

## Introduction

Coronavirus disease 2019 (COVID-19), caused by the infection of SARS-CoV-2 can develop into severe pneumonia, produce an immune storm and neurodegenerative disorders [1, 2]. In most cases, SARS-CoV-2 entry into host cells is mediated by the interaction of the viral spike (S) protein with the angiotensin-converting enzyme 2 (ACE2) receptor in respiratory epithelial cells [3]. Since the S protein of SARS-CoV-2 participates in the entry of most host cells, the development of neutralizing antibodies and vaccines mostly targets the S protein [4, 5]. In addition to ACE2-mediated viral infection, other receptors have also been found to promote the entry of SARS-CoV-2 into host cells, such as AXL tyrosine kinase receptor (a receptor tyrosine kinase of the TYRO3-AXL-MER family), neuropilin-1, and heparin sulfate [6–9]. Inhibiting AXL kinase activity has antiviral effects [10]. At present, a variety of AXL inhibitors are being studied in different stages of clinical trials. Among these, the small-molecule inhibitor BGB324 was the first AXL kinase inhibitor to be developed [11] and clinically tested [12] against COVID-19 (ACCORD-2-001). However, AXL small-molecule inhibitors may inhibit other kinases producing off-target effects. Clinical trials have shown that AXL inhibitors cause adverse effects [13, 14]. The search for drugs that inhibit AXL is an option.

Quercetin is a polyphenolic flavonoid widely found in fruits and vegetables and is known to have various pharmacological activities [15]. Recent studies have shown that quercetin suppresses the transcriptional expression of AXL [16] and IL-6/STAT3 signaling in the type II human pneumocyte cell line A549 [17]. The results of molecular docking studies indicated that quercetin interacts with the S and 3CLpro proteins of SARS-CoV-2 [18, 19]. Quercetin improves symptoms during the early stages of SARS-CoV-2 infection and prevents severe COVID-19 [20]. Therefore, quercetin appears to be an ideal candidate for further exploration as an agent to combat COVID-19. However, quercetin has low water solubility, oral bioavailability [21, 22], and additionally decreased human bronchial epithelial BEAS-2B cell viability in a dose- and time-dependent manner [23, 24]. Thus, new carriers and routes of quercetin administration need to be developed.

As ACE2 and AXL are known to be involved in SARS-CoV-2 entry into host cells, targeting both ACE2 and AXL may effectively inhibit viral entry into cells [25]. However, the development of agents that simultaneously target ACE2 and AXL remains challenging. Organic, inorganic, and metal nanoparticles can reportedly be used for the *in vivo *delivery of therapeutic molecules [26]. Polymers and lipid nanoparticles, such as polyglycolic acid (PGA) and polylactic acid (PLA), have better biocompatibility and biodegradable properties, and their copolymer [poly(lactic-co-glycolic acid)], has been approved by the FDA for *in vivo *delivery [27]. In this study, we used PLGA nanoparticles (NPs) to prepare NPs incorporated with quercetin (NP-Q), ACE2-containing cell membranes (CM-NPs), or both quercetin- and ACE-2-containing cell membranes (CM-NP-Q). These nanoparticles were then tested for physicochemical characterization [28], cytotoxic activity and their ability to neutralize or inhibit viral infection.

## Results

### Preparation and characterization of nanoparticles encapsulated with quercetin and/or coated ACE2-containing cell membranes

**Fig. 1:**
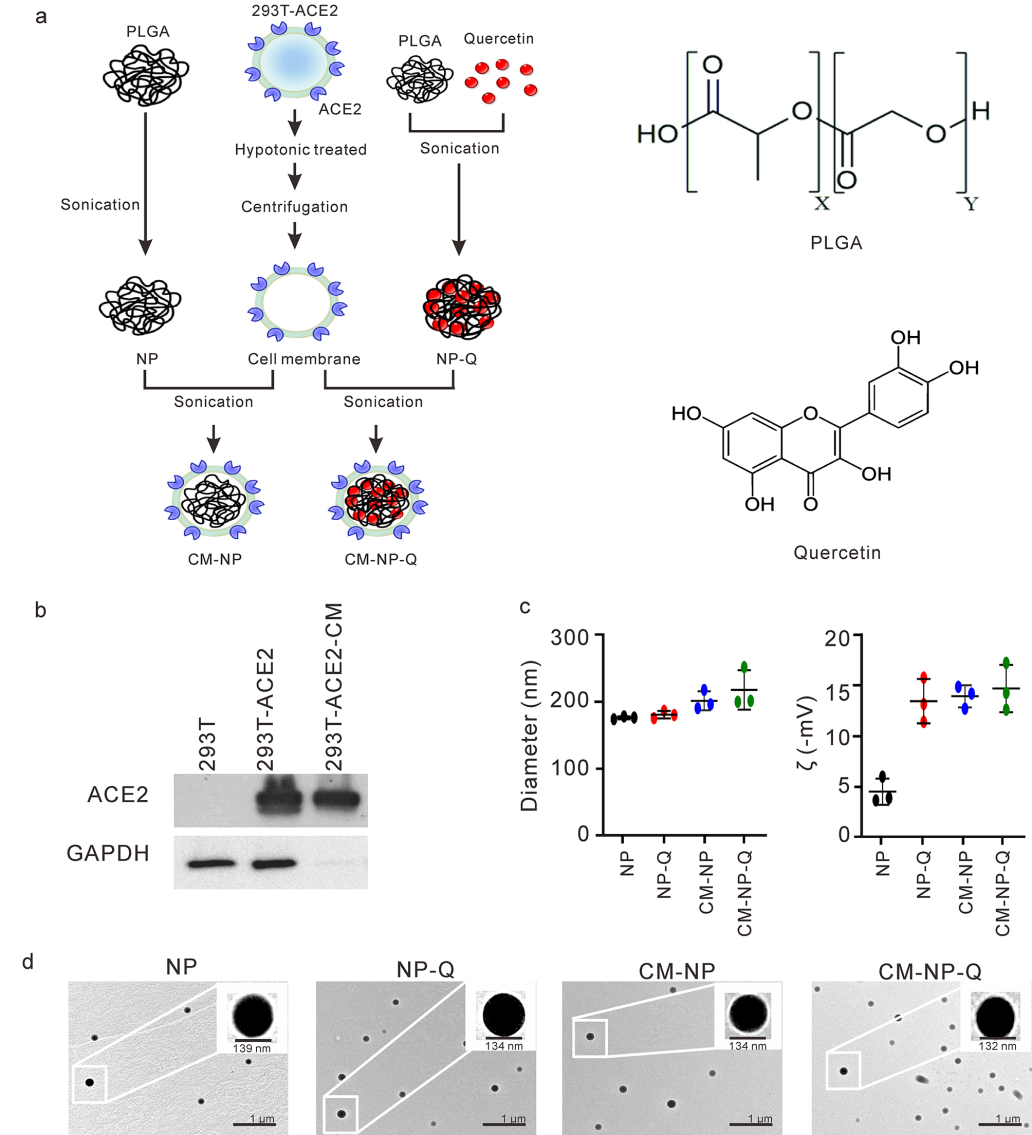
Preparation and physical characterization of nanoparticles incorporated with quercetin and/or cell membranes. (A). Schematic diagram for the preparation of nanoparticles encapsulated with quercetin (NP-Q), cell membranes (CM-NP), or both quercetin and cell membranes (CM-NP-Q). The chemical structures of PLGA and quercetin are shown at the right. (B). Western blot analysis of the cell membrane prepared from 293T-ACE2 cells. The extracted cell membrane (293T-ACE2-CM) and the total cell lysates from 293T-ACE2 cells were analyzed for the presence of ACE2 by Western blotting. GAPDH served as a cytoplasmic marker. (C). The diameter and zeta potential (ζ) of the nanoparticles were determined by laser-scattering method. The data shown here are the means of three independent experiments ± SD. (D). Transmission electron micrographs of the nanoparticles. Images in the inset are magnified nanoparticles. Scale bar = 1 μm

To explore the feasibility of encapsulating nanoparticles with quercetin and/or ACE2-containing cell membranes to combat SARS-CoV-2 infection, we prepared nanoparticles encapsulated with quercetin and/or ACE2-containing cell membranes, as shown in Fig. 1A. The nanoparticles encapsulated with quercetin (NP-Q) were found to have a 98.53%± 0.37 entrapment of quercetin as determined by HPLC analysis. The ACE2-containing cell membrane was prepared from 293-ACE2 cells as shown in Fig. 1B. This cell membrane preparation was used to coat nanoparticles (NPs) and nanoparticles encapsulated with quercetin (NP-Q) to obtain cell membrane nanoparticles (CM-NPs) and cell membrane nanoparticles encapsulated with quercetin (CM-NP-Q). The diameter and surface charge of these nanoparticle preparations were determined by dynamic laser scattering and are shown in Fig. 1C and Supplementary Table 1. The average diameter of NP, NP-Q, CM-NP or CM-NP-Q is approximately 200 nm, and all the nanoparticles have a negative surface potential. The polydispersity index (PDI) of all tested nanoparticles was below 0.31, demonstrating size uniformity. We further used transmission electron microscopy (TEM) and scanning electron microscope (SEM) to confirm the spherical and uniform size of these nanoparticle preparations. CM-NP and CM-NP-Q exhibit a core-shell structure, and a bright coating cell membrane is visible around the polymer particles (Fig. 1D and Supplementary Fig. 1). There was no significant changes in the hydrodynamic size of CM-NP-Q incubated in phosphate-buffered saline (PBS) or culture medium for 3 h at 37 °C (Supplementary Fig. 2A). Because all of the nanoparticles are smaller than 250 nm and negatively charged, they should not elicit activation of the complement system or phagocytosis by macrophages [29, 30].

**Fig. 2:**
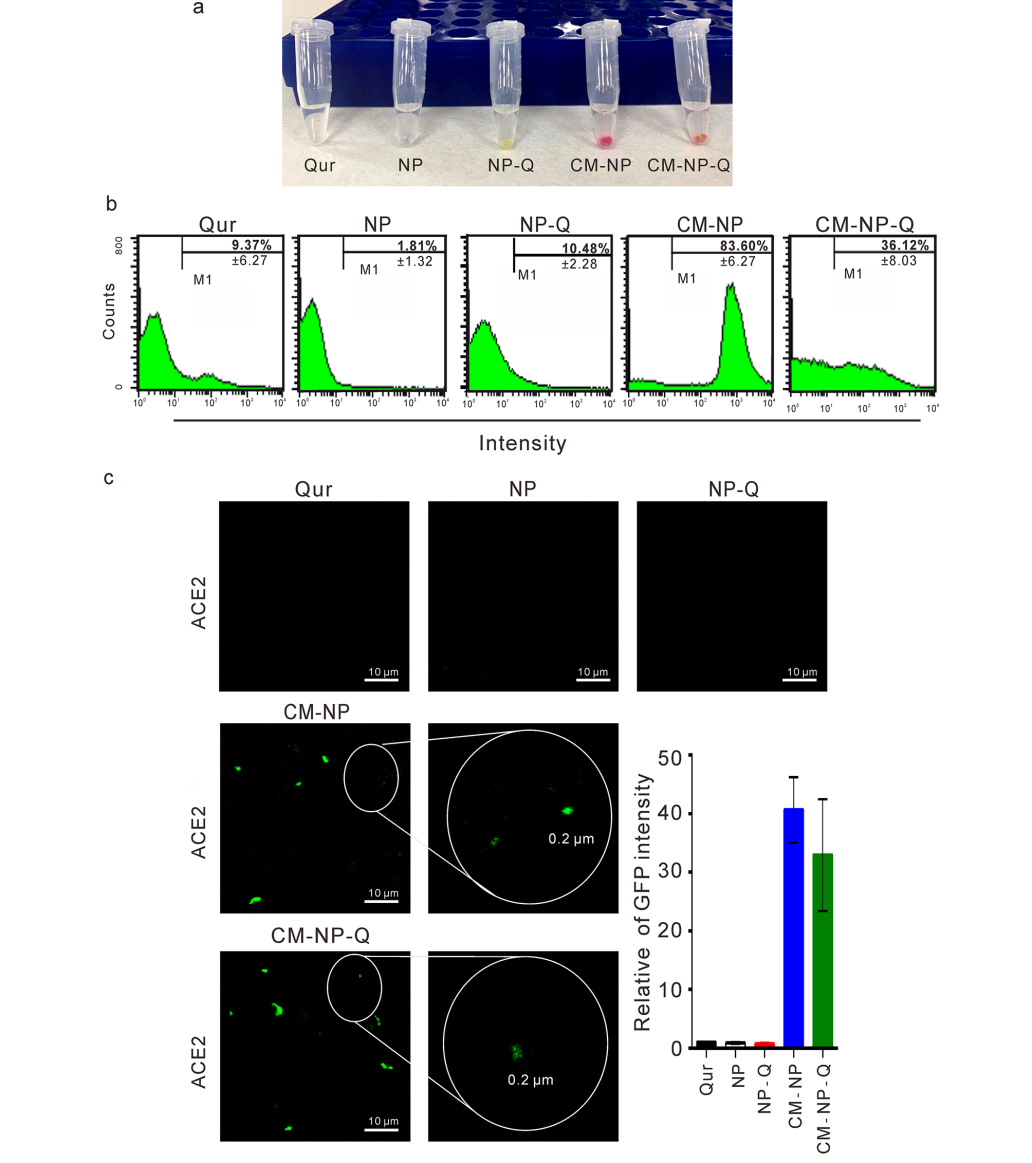
Detection of ACE2-cell membranes in the nanoparticles. (A). Images of the Ponceau S staining test with nanoparticles. Flow cytometry (B) and immunofluorescence (C) analysis of ACE2 expression in the cell membrane nanoparticles. The intensity of GFP from (C) was analyzed using the MetaMorph software. The statistical data shown in (B) and (C) are the means of three independent experiments ± SD. (C) Scale bar = 10 μm

To confirm that CM-NP and CM-NP-Q nanoparticles encapsulated the cell membrane, protein staining with Ponceau S was used to detect the presence of protein from 293-ACE2 cell membrane. As shown in Fig. 2A, CM-NP and CM-NP-Q stained positive for the presence of protein but not NP or NP-Q. To address whether ACE2 protein is present in the CM-NP and CM-NP-Q, nanoparticles were treated with ACE2-Alexa Fluor 647 antibody and then analyzed by flow cytometry (Fig. 2B) and confocal microscopy (Fig. 2C). As shown in Fig. 2B, a significantly higher percentage of ACE2-positive particles was detected in CM-NP (83.60%±6.27) and CM-NP-Q (36.12%±8.03) than in quercetin (9.37%±6.27), NP (1.81%±1.32), or NP-Q (10.48%±2.28). However, because quercetin may interfere with ACE2-positive signaling in CM-NP-Q cells due to the fluorescence spectrum of quercetin having a peak at 505 nm [31], we also employed confocal microscopy to detect the presence of ACE2 in nanoparticles. As shown in Fig. 2C, ACE2 was detected only in CM-NP and CM-NP-Q but not in quercetin, NP, or NP-Q. These results indicate that CM-NP and CM-NP-Q successfully coated the 293T-ACE2 cell membrane. To evaluate the long-term stability of CM-NP-Q, the content of quercetin in CM-NP-Q stored at 4 °C was analyzed over a period of three months. There was a 7.32%±1.52 decrease in quercetin content after 3 months of storage. No significant change was detected in the diameter and surface charge. In addition, the stability of quercetin in CM-NP-Q and NP-Q was assayed by the release of quercetin from nanoparticles incubated in PBS at 37 °C (Supplementary Fig. 2B). The quercetin in NP-Q was continued to release slowly and a total of 0.4% was found to be released at 48 h. In contrast, the release of quercetin from CM-NP-Q appeared to peak at 12 h with a total release of only 0.2%, suggesting that quercetin was more stabilized in the CM-NP-Q.

Lastly, differential scanning calorimetry (DSC) thermograms was used to confirm the incorporated quercetin and/or cell membranes in the nanoparticles. As shown in Supplementary Fig. 2C, the DSC curve of NP showed an endothermic peak at 51 °C, which is due to the glass transition temperature (Tg) of PLGA [32]. For NP-Q, the DSC curve showed two endothermic peaks near 49 °C and 120 °C, corresponding to the Tg of NP and quercetin (water loss peak at 130 °C) [33], respectively. The DSC profile of CM-NPs displayed a melting range between 40 and 130 °C and a small endothermic peak near 49 °C, reflecting the cell membrane melting between 40 and 130 °C and containing the Tg of NP. For CM-NP-Q, the DSC curve showed a melting range between 40 and 130 °C (cell membrane melting range) and two small peaks around (48 °C and 120°), indicating that the CM-NP-Q incorporated with quercetin and cell membranes.

### Cellular uptake of nanoparticles

**Fig. 3:**
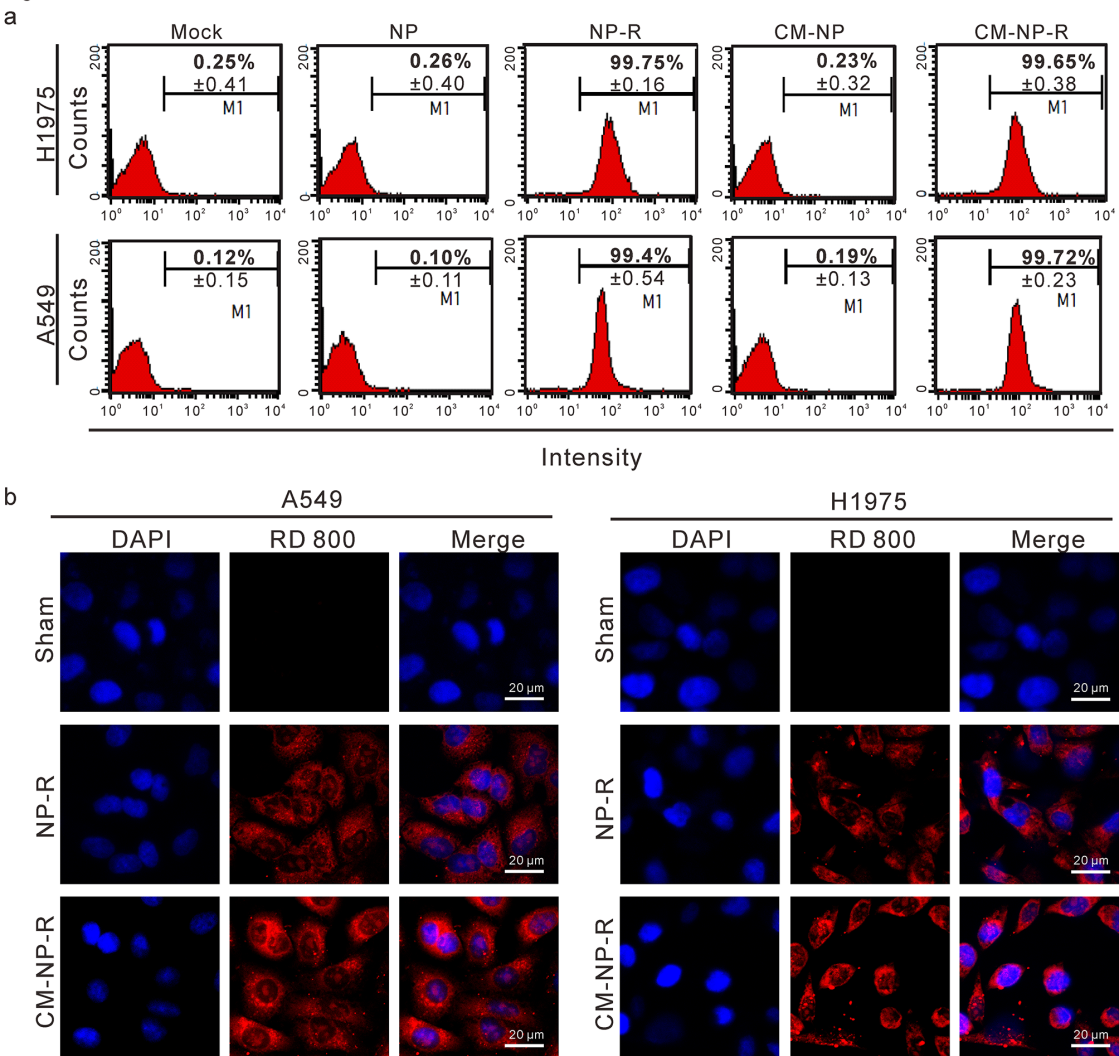
The cellular uptake of nanoparticles by lung cell lines. H1975 and A549 cells were incubated with Rhodamine 800-labeled nanoparticles for 24 h. The cellular uptake and subcellular localization of nanoparticles in the cells were examined by flow cytometry (A) and confocal laser scanning microscopy (B), respectively. The blue color is the DAPI staining of nuclei, while the red color is the signaling of Rhodamine 800 (RD 800). Scale bar = 20 μm. The statistical data shown in (A) are the means of three independent experiments ± SD.

To address whether cells may take up nanoparticles, the NPs and CM-NPs were labeled with rhodamine 800 and incubated with H1975 (an AXL expressing cell line) [34] and A549 cells (a type II human pneumocyte cell line, which the SARS-CoV-2 can target) [35] for 24 h. Flow cytometry analysis showed that 99% of the H1975 and A549 cells incubated with NP-rhodamine (NP-R) or CM-NP-rhodamine (CM-NP-R) exhibited intracellular fluorescence signals (Fig. 3A). Confocal microscopy analysis further revealed that red fluorescence of NP-R and CM-NP-R were detected in both the cytoplasmic and nuclear regions of H1975 and A549 cells, but not at the cell membrane (Fig. 3B).

### Effects of NP-Q and CM-NP-Q nanoparticles on the cytotoxic activity and inhibition of AXL

**Fig. 4:**
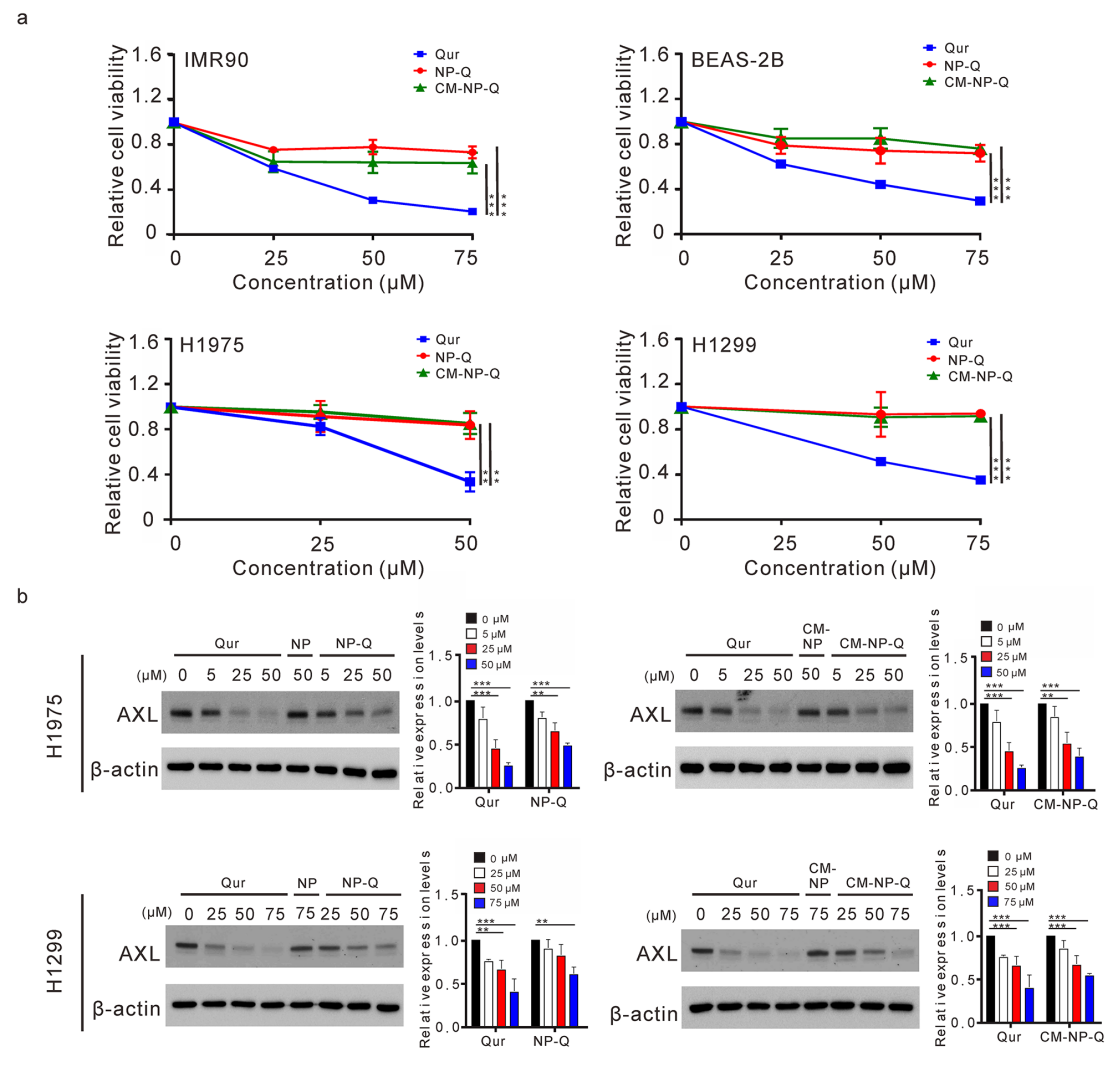
Effects of nanoparticles on cytotoxic activity and AXL suppression in lung cell lines. (A). IMR90, BEAS-2B, H1975 and H1299 cells were incubated with free quercetin (Qur), NP-Q or CM-NP-Q for 48 h, and cell viability was determined by MTT assay. (B). The cells were treated with nanoparticles for 24 h, and the AXL expression level was detected by Western blot. β-actin served as the loading control and the relative expression levels of AXL were quantified. The data shown here are the means of three independent experiments ± SD. **p < 0.01 and ***p < 0.001

To address whether quercetin coated on NP-Q and CM-NP-Q may inhibit AXL and exert cytotoxic activity, cells (BEAS-2B, IMR90, H1299 and H1975) were treated with different concentrations of quercetin, NP-Q and CM-NP-Q to examine their effects on cell viability and the expression of AXL. As shown in Fig. 4A, treatment of cells with free quercetin produced significant cytotoxic activity. However, there were no significant cytotoxic effects of NP-Q and CM-NP-Q in all of the treated cells. The effects of quercetin, NP-Q and CM-NP-Q on the expression of AXL in H1299 and H1975 cells are shown in Fig. 4B. While free quercetin greatly inhibited the expression of AXL (Fig. 4B), both NP-Q and CM-NP-Q were found to be able to inhibit the expression of AXL in the treated cells, although at a lesser activity.

### Effect of nanoparticles on the neutralization of extracellular pseudo virus

**Fig. 5:**
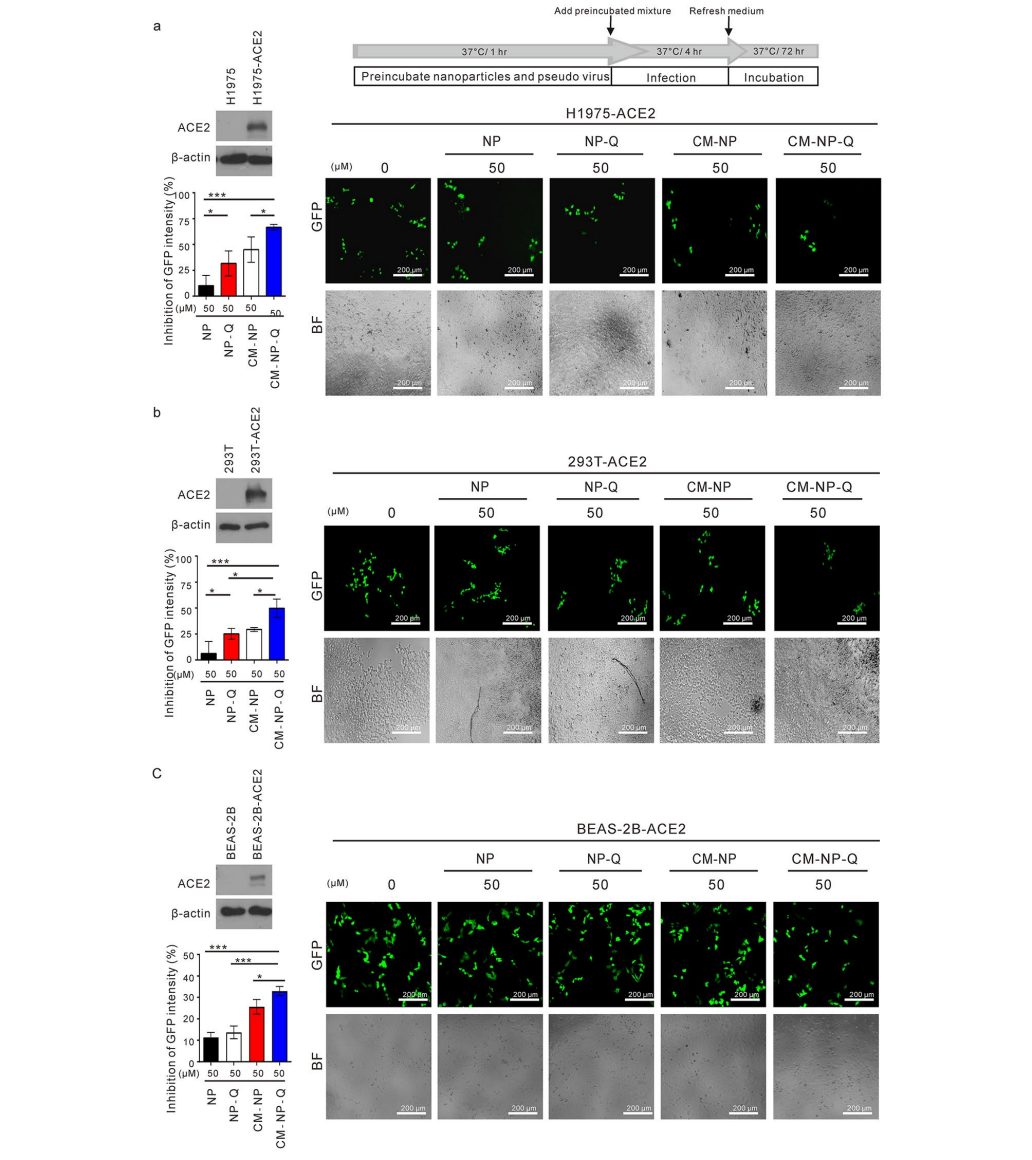
Effects of nanoparticles on the neutralization of extracellular pseudo virus. The nanoparticle preparations were incubated with pseudotyped SARS-CoV-2 lentivirus expressing green fluorescent protein (GFP) at 37 °C for 1 h. The mixtures were then added to (A) ACE2-expressing H1975 cells (H1975-ACE2), (B) 293T cells (293T-ACE2), or (C) BEAS-2B cells (BEAS-2B-ACE2). Assay for viral infection was done by detecting the GFP-expressing cells as described in the Materials and Methods. The overexpression of ACE2 in H1975-ACE2 cells, 293T-ACE2 cells, and BEAS-2B-ACE2 cells was determined by Western blot and shown in the left panels. The expression of GFP was evaluated using microscopy. BF, bright field. The percent inhibition (%) was calculated as 1-(GFP signal in the presence of nanoparticles)/(GFP signal in the absence of nanoparticles) × 100. Data points represent the mean of three independent experiments ± SD. *p < 0.05 and ***p < 0.001. Scale bar = 200 μm

To evaluate whether nanoparticles may react with the virus and prevent its infection ability, we established a pseudotyped lentivirus expressing S protein and green fluorescence (GFP) for the pseudotype SARS-CoV-2 infection assay. In the neutralization assay, the nanoparticles (NP, NP-Q, CM-NP, or CM-NP-Q) were incubated with the pseudo virus for 1 h before being added to H1975-ACE2, 293T-ACE2, and BEAS-2B-ACE2 cells. The media were replaced after 4 h, and incubation continued for an additional 72 h. The expression of GFP in the cells was detected by fluorescence microscopy. As shown in Fig. 5A, B and C, preincubating the pseudo viruses with NP-Q, CM-NP and CM-NP-Q reduced pseudo virus infection in H1975-ACE2, 293T-ACE2, and BEAS-2B-ACE2 cells. NP-Q, CM-NP and CM-NP-Q were found to be able to block pseudo virus infection.

### Effects of nanoparticles on pseudo virus and SARS-CoV-2/NTU13 infection

**Fig. 6:**
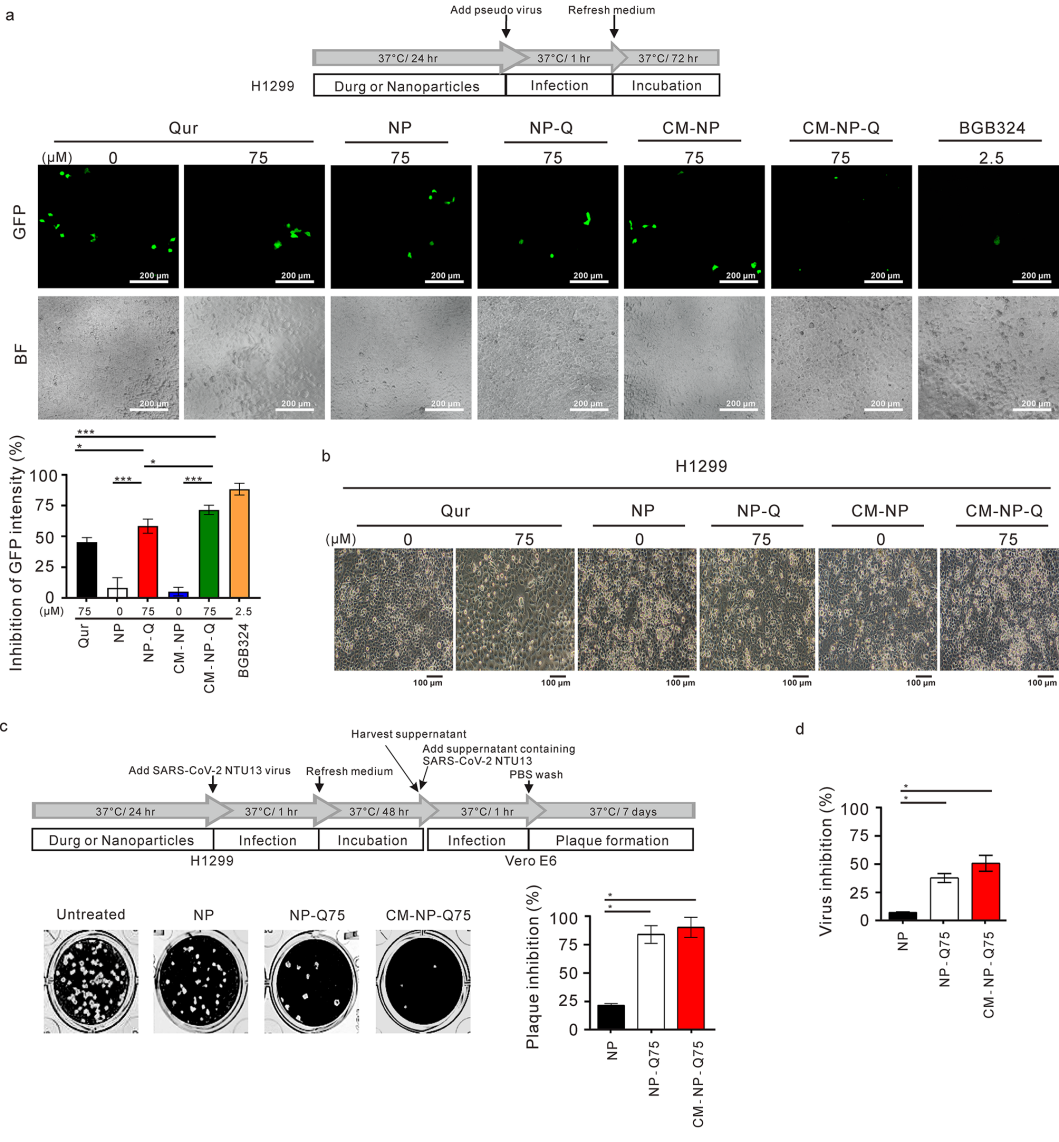
Effect of nanoparticles on the inhibition of pseudo virus and SARS-CoV-2 infection. H1299 cells were treated with nanoparticles or BGB324 for 24 h before being infected with pseudotyped lentivirus or SARS-CoV-2/NTU13. After 1 h of infection, the virus-containing media was removed and replaced with fresh media. (A) For cells infected with pseudotyped lentivirus, the GFP expression was evaluated after 72 h using microscopy images (upper panel). The percent inhibition (%) was calculated as 1-(GFP signal in the treated sample/ (GFP signal in the untreated control) × 100. BF, bright field. Scale bar = 200 μm. Data points represent the mean of three independent experiments ± SD. (B) Images of H1299 cells infected with SARS-CoV-2/NTU13 for 48 h. Scale bar = 100 μm. (C) Plaque formation assay was performed with H1299 cells infected with SARS-CoV-2/NTU13. The percentage of inhibition was calculated as 1-(VD/VC) × 100, where VD and VC refer to the virus titer in the treated and untreated of the test compound, respectively. The data points represent the means of three independent experiments ± SD. (D) Viral yield reduction assay was performed using qRT-PCR to detect the amount of viral RNA. The percentage of inhibition was calculated as 1-(VD/VC) × 100, where VD and VC refer to the virus titer in the treated and untreated of the test compound, respectively. The data points represent the means of three independent experiments ± SD. *p < 0.05 and ***p < 0.001

Next, the ability of nanoparticles to inhibit AXL-mediated SARS-CoV-2 infection was examined in the AXL-mediated SARS-CoV-2 infection cell line H1299 [6]. The AXL-specific inhibitor BGB324 was confirmed in its ability to block virus infection [9]. The results for the inhibition of pseudovirus study were shown in Fig. 6A. As expected, the AXL-specific inhibitor BGB324 was confirmed to block virus infection as previously reported by others [12]. While CM-NP did not inhibit AXL-mediated pseudo virus infection, CM-NP-Q produced a stronger inhibition of pseudo virus infection than free quercetin and NP-Q at 75 µM. To further confirm the inhibitory effects of nanoparticles was *in vivo*, we examined the effects of nanoparticles on the infection by SARS-CoV-2/NTU13 virus. A morphological examination of cells infected with SARS-CoV-2/NTU13 at a MOI of 0.5 for 48 h indicated that only the cells treated with free drugs displayed low cell density (Fig. 6B). The results of plaque and viral yield reduction assay were shown in Fig. 6C and D, respectively. Both NP-Q and CM-NP-Q significantly inhibited virus infection.

**Effect of nanoparticles on** *in vivo* **safety**.

**Fig. 7:**
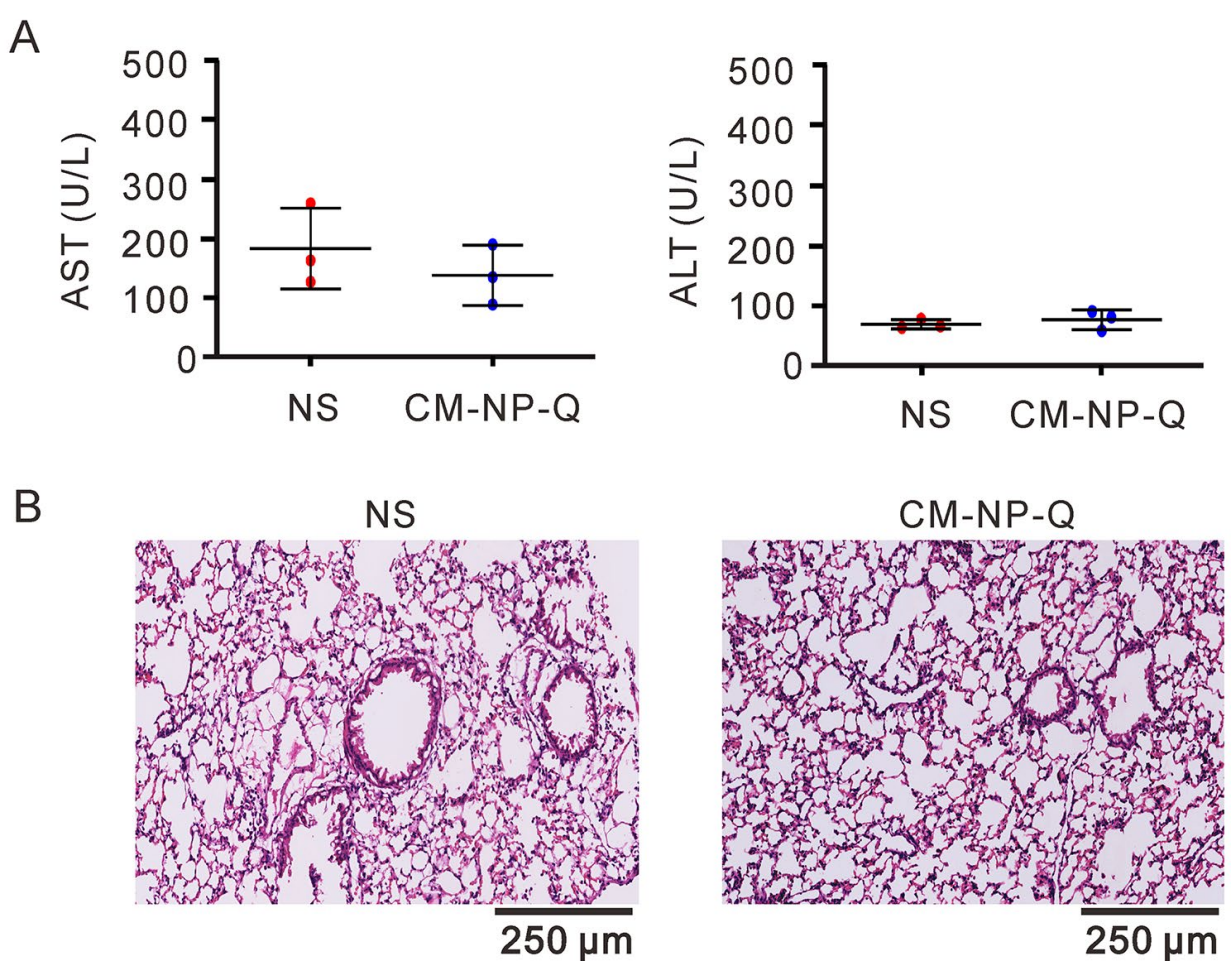
Effects of nanoparticles on biosafety in mice. BALB/c male mice were treated intratracheally with 50 µL of 0.9% normal saline or 10 µM CM-NP-Q in 0.9% normal saline (NS) for 24 h (n = three/group). (A) The serum levels of aspartate transaminase (AST) and alanine transaminase (ALT) were analyzed as described in Materials and Methods. The data points represent the means of three mice ± SD. (B) Histological images of representative lung section. Scale bar = 250 μm

After observing the inhibitory effects of nanoparticles on virus infection (Figs. 5 and 6), we further examined the *in vivo* toxic effects of nanoparticles in mice. Mice were intratracheally treated with 50 µL of 0.9% normal saline or 10 µM CM-NP-Q in 0.9% normal saline for 24 h. As shown in Fig. 7A, there was no significant difference in the serum levels of serum aspartate transaminase (AST) and alanine transaminase (ALT) between the control and CM-NP-Q-treated mice, suggesting that there is no significant liver damage caused by the nanoparticles. Histological examination of lung by hematoxylin and eosin (H&E) staining also revealed that there are no alterations in the lung of nanoparticle-treated mice (Fig. 7B).

## Discussion

In this study, we developed nanoparticles incorporated with quercetin and ACE2-membrane as a novel ACE2 and AXL-targeting therapy for COVID-19 treatment. The PLGA nanoparticles (NP) were used to prepare NP encapsulated with quercetin (NP-Q), ACE2-containing cell membrane (CM-NP), or with both quercetin and ACE-2 containing cell membrane (CM-NP-Q). These nanoparticle preparations were smaller than 250 nm and negatively charged. The NP-Q was found to have 98.53%± 0.37 entrapment of quercetin as determined by HPLC analysis. We have shown that both CM-NP and CM-NP-Q have successfully coated the 293T-ACE2 cell membrane (Fig. 2). These nanoparticles can be developed into inhaler sprays to be directly inhaled into the respiratory system, increasing its therapeutic effect on the respiratory system and reducing side effects. Therefore, these nanoparticle preparations should be ideal for targeting ACE2 and AXL in the COVID-19 treatment.

We have observed that the activity of quercetin encapsulated onto NP-Q and CM-NP-Q appeared to differ from that of free quercetin. As shown in Fig. 4, treating the cells with free quercetin produced significant cytotoxic activity in the treated bronchial and lung cells, but treating with NP-Q and CM-NP-Q did not. While the free quercetin greatly inhibited the expression of AXL, both NP-Q and CM-NP-Q were found to have lower activity in inhibiting the expression of AXL. The decreased activity of quercetin in NP-Q and CM-NP-Q may be due to the lipophilicity of quercetin that could have high affinity with NP [36]. The release quercetin from a solid lipid microparticles or PLGA nanoparticles is slow and constant via diffusion mechanism [37, 38]. The release of quercetin from PLGA nanoparticles is enhanced in the acidic conditions [39]. However, the potential of using this approach to increase drug release remains to be explored.

We have examined the ability of nanoparticle preparations to neutralize extracellular pseudo-SARS-CoV-2 virus. As expected, nanoparticles coated with ACE2-containing membrane (CM-NP and CM-NP-Q) were found to be able to neutralize the ACE2-mediated virus infection (Fig. 5). However, much to our surprise, we observed that the NP-Q was also able to neutralize the virus infection. Molecular docking analysis has indicated that quercetin can interact with the S protein and 3CLpro protein of SARS-CoV-2 [18, 19]. This interaction, therefore, may explain why NP-Q also has the ability to neutralize the virus infection. To further understand the drug effects for COVID-19 treatment, there is a need to develop 3D multi-cell interactions models to testing [40].

Lastly, we employed H1299 cells to examine the ability of the nanoparticle preparations to inhibit AXL-mediated SARS-CoV-2 infection. As shown in Fig. 6, we observed that CM-NP-Q was more effective than free quercetin and NP-Q at inhibiting pseudo virus and SARS-CoV-2/NTU13 infection. Therefore, development of inhaler sprays containing CM-NP-Q should be useful for the treatment of COVID. Since CM-NP-Q had little cytotoxic activity (Fig. 4A) and is to be inhaled directly into the respiratory system, it should produce little side effects while inhibiting viral infection. However, large-scale expansion of consistent ACE2-containing cell membrane may represent a major challenge to produce CM-NP-Q in clinical application. Although purified ACE2 protein represents a suitable substitute to replace ACE2-containing membrane, it remains to be investigated if ACE2 protein may be encapsulated into nanoparticles to produce the same inhibitory effects as CM-NP-Q. In addition, the metabolic mechanism and safety of ACE2-nanoparticles *in vivo *as well as their effects on immunogenic and inflammatory responses require further investigations.

## Conclusions

**Fig. 8:**
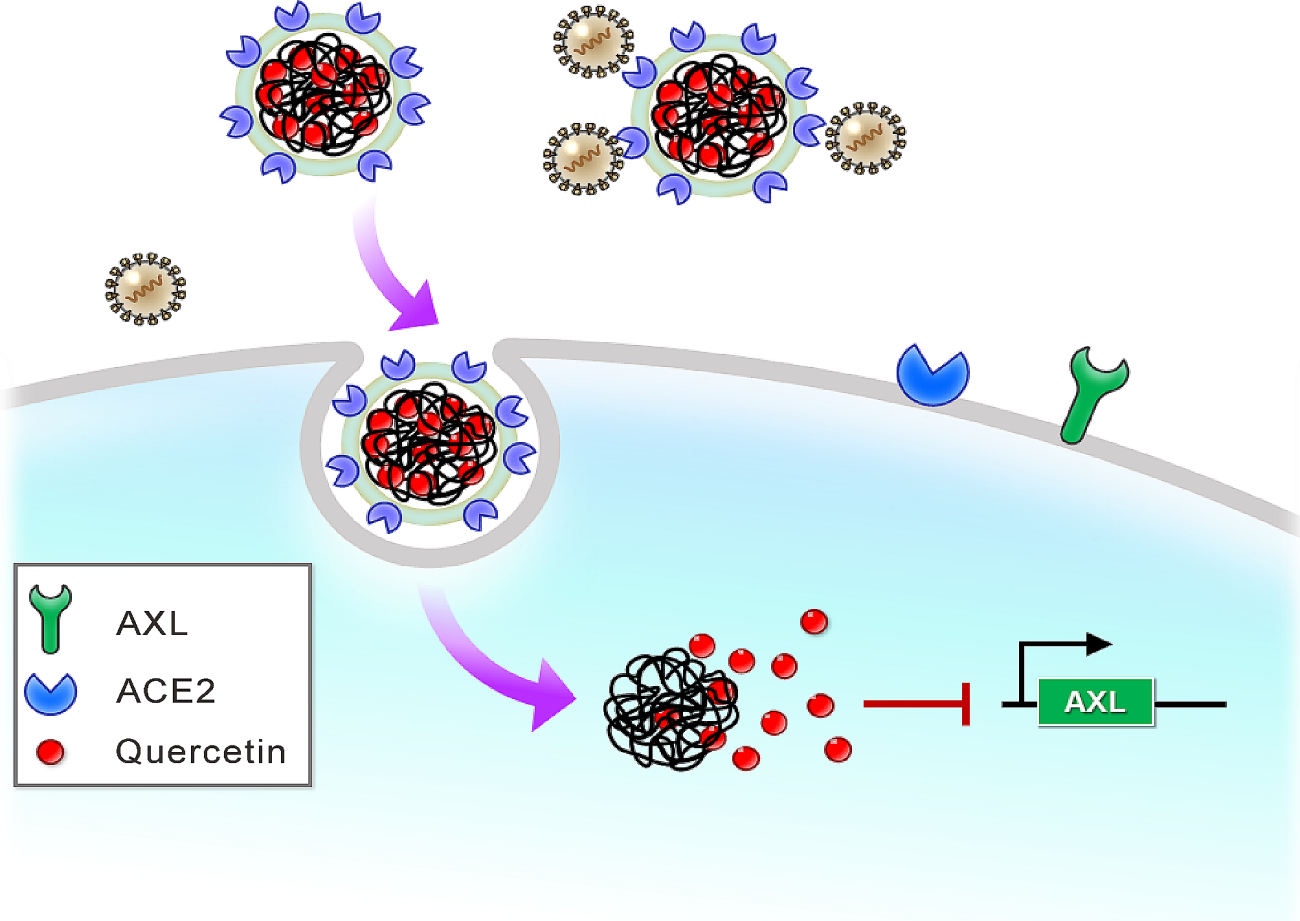
Model of CM-NP-Q in neutralizing and inhibiting virus infection

In summary, the biomimetic nanoparticles coated with ACE-2 membrane and quercetin (CM-NP-Q) showed the strongest antiviral activity, which may be attributed to its ability to neutralize and inhibit SARS-CoV-2 infection, as shown in the proposed model (Fig. 8).

## Materials and methods

### Culture media and reagents

Fetal bovine serum (FBS) and culture media were purchased from Life Technologies (Grand Island, NY, USA). Quercetin (CAS 6151-25-3), PLGA [poly (lactic-co-glycolic acid)] and other chemicals were purchased from Sigma-Aldrich. BGB324 was purchased from Selleck.

### Cell lines

A549, H1299, H1975, BEAS-2B and 293T cells were obtained from the American Type Culture Collection (Manassas, VA, USA). Normal human lung fibroblasts (IMR90) were purchased from the Food Industry Research and Development Institute in Taiwan. The BEAS-2B is a normal human bronchial epithelial cell line. The 293T-ACE2, H1975-ACE2, and BEAS-2B-ACE2 strains were constructed by transduction with lentivirus encoding full-length ACE2 as described in a previous study [41]. The RPMI cell culture medium used for A549, H1299, H1975, and 293T cell lines contained 10% FBS, 1% Pen-Strep-Ampho Sol., and 1% sodium pyruvate. IMR90 and BEAS-2B cells were cultured in Dulbecco’s modified Eagle’s medium supplemented with 10% FBS and 1% Pen-Strep-Ampho Sol. All the cell lines were verified by STR analysis [16].

### Cell membrane preparation

Cell membranes of 293T-ACE2 cells were obtained according to a previous study [42]. In brief, 293T-ACE2 cells were suspended in cold hypotonic lysis buffer (Tris-HCl 30 mM pH = 7.5, D-mannitol 225 mM, sucrose 75 mM, EGTA 0.2 mM and a cocktail of protease and phosphatase inhibitors) and homogenized through 21-gauge syringe needles. The homogenates were centrifuged at 20,000 × g for 25 min at 4 °C. The supernatant was collected and centrifuged again at 100,000 × g for 35 min. After centrifugation, the cell membranes in the pellets were collected and suspended in 0.2 mM EDTA. The concentration of the cell membranes was quantified by using a BCA protein assay kit (Pierce). The cell membranes were stored at -80 °C.

**Preparation of nanoparticles incorporated with quercetin and/or cell membrane.**

The nanoparticle cores (NP) and nanoparticles coated with quercetin or rhodamine 800 (a fluorescence dye, for track of cellular uptake of nanoparticles) were prepared by dissolving 50 mg of PLGA (for NP) and 50 mg of PLGA plus 5 mg of quercetin (for NP-Q) or 1 mg of rhodamine 800 (for NP-R), respectively, in 4 ml of dichloromethane (DCM) to form an oil phase. The oil phase was added to 12 ml of 2.5% polyvinyl alcohol, and then the mixture was emulsified by a probe-type sonicator for 5 min at 35 W on ice. The DCM in the mixture was evaporated with magnetic stirring overnight. Then, the resulting mixture was centrifuged at 10,000 rpm for 10 min. The pellets were washed three times with phosphate-buffered saline (PBS) and suspended in 10 ml of double-distilled water. To prepare the NPs, NP-Q and NP-R coated with cell membrane (CM), 500 µg of cell membrane was mixed with 1 mg of the NP, NP-Q, or NP-R and sonicated for 5 min at 35 W to make CM-NP, CM-NP-Q, and CM-NP-R, respectively. The concentrations of all the nanoparticle preparations were expressed in terms of the NPs present in the preparations.

### Encapsulation efficiency of quercetin

The NP-Q preparations were centrifuged at 15,000 g at 4 °C for 30 min. The supernatant and the precipitate were then analyzed by high-performance liquid chromatography (HPLC) to determine the percentage of quercetin entrapped by NPs [43]. The wavelength of the ultraviolet-visible detector was set at 256 nm.

### Characterization of cell membrane nanoparticles

The methodologies for assessing nanoparticle size, zeta potential, drug release, and stability were performed as described previously [43]. In brief, the nanoparticles incorporated with quercetin and/or the cell membrane were diluted 100-fold with double-distilled water, and the hydrodynamic size (*z*-average), PDI and surface charge were measured by laser-scattering method (Nano ZS90, Malvern). The stability of cell membrane nanoparticles was evaluated by determining size changes following incubation in PBS or culture medium in sealed vials at 37 °C for 3 h. For drug release test, the CM-NP-Q or NP-Q were loaded into dialysis bag (MWCO 12,000–14,000 Da, SERVA, Germany) and placed in 100 ml of PBS at 37 °C. At various time points, the incubation buffer was taken and replaced with fresh buffer. The amount of quercetin in incubation buffer was determined by HPLC method [43].

### Transmission electron microscopy and scanning electron microscopy

The size and morphology of nanoparticle preparations were studied by scanning electron microscopy (SEM, SU-8220, Hitachi, Tokyo, Japan) and transmission electron microscopy (TEM, HT-7800, Hitachi, Tokyo, Japan). For TEM imaging, 500 µl of nanoparticles was deposited onto a copper grid. After five minutes, the samples were stained using phosphotungstic acid 0.5%, and the morphology of these nanoparticles was imaged by the TEM operating at 100 kV and processed using Quartz PCI software. For SEM analysis, the samples were dried and coated with gold. The images were taken at an accelerating voltage of 10 kV and at different magnifications [44].

### Differential scanning calorimetry (DSC)

DSC thermograms were assayed on a Differential Scanning Calorimeter (Perkin Elmer, USA) for spray-dried samples and their pure materials [43]. In brief, after placing the sample on the flat-bottomed aluminum plate, the scanning temperature was set for 0–200 °C, with the heating rate at 10 °C/min under nitrogen atmosphere, and the flow rate was 20 ml/min. Data relevant to the observed thermal events were reported as peak temperatures.

### Cellular uptake of nanoparticles

The nanoparticles containing fluorescent rhodamine 800 (NP-R or CM-NP-R), NP, or CM-NP were incubated with the cells at 37 °C for 24 h [45]. Cells were trypsinized and washed with PBS. A total of 10,000 cells were analyzed by flow cytometry (BD caliber)) using excitation wavelength at 633 nm. An LSM 700 laser scanning confocal microscope (Carl Zeiss) was employed to image the cellular uptake.

### Detection of ACE2-cell membranes in the nanoparticles

The nanoparticle preparations (CM-NP and CM-NP-Q) were incubated with Alexa 647-labeled anti-ACE2 antibody (Santa Cruz Biotechnology) for 24 h and then fluorescent secondary antibody [46]. The presence of ACE2-cell membranes in the nanoparticles was analyzed by confocal microscopy and flow cytometry. The MetaMorph software was used to examine GFP intensity (MetaMorph Inc., Nashville, TN, USA) [46].

### Cell viability test

Cell viability was detected by MTT (3-(4,5-dimethylthiazol-2-yl)-2,5-diphenyltetrazolium bromide) assay according to the manufacturer’s instructions (Sigma‒Aldrich, USA) [47].

### Neutralization and inhibition of SARS-CoV-2 pseudotyped lentivirus infection

The SARS-CoV-2 spike carrying the D614G mutation pseudotyped lentivirus was produced by transient transfection of HEK293T cells with pLAS2w.EGFP.pPuro, pMDG and pCMV-DR8.91 as previously described [41]. For the neutralization assay, the nanoparticle preparations were incubated with pseudo virus at 37 °C for 1 h. The mixtures were added to ACE2-expressing H1975 cells (H1975-ACE2), 293T cells (293T-ACE2) or BEAS-2B cells (BEAS-2B-ACE2) and incubated for an additional 4 h. Then, the pseudo virus-containing media were replaced with fresh media. After an additional 72 h, the cells infected with the pseudo virus were detected using an Olympus IX73 Microscope Imaging System (Olympus, Japan). Cells expressing green fluorescent protein (GFP) are considered to be infected with SARS-CoV-2 pseudotyped lentivirus [41]. For the inhibition studies, H1299 cells were treated with the drug or nanoparticle preparations for 24 h before being infected with virus. After 1 h of infection, the virus-containing media was removed and replaced with fresh media. After incubating for an additional 72 h, the infection efficiency was evaluated by detecting the GFP expression using microscopy. The percent inhibition was calculated by 1-(D/C), in which D and C refer to the presence and absence of quercetin, respectively.

### Plaque and viral yield reduction assays

The virus strain used in this study was SARS-CoV-2/NTU13/TWN/human/2020 (Accession ID EPI_ISL_422415). Isolation of SARS-CoV-2/NTU13 was performed as described previously [41]. The study involving SARS-CoV-2/NTU13 was approved by the National Taiwan University Hospital Research Ethics Committee (202002002RIND), and written informed consent was obtained from all participants. In the plaque reduction assay, H1299 cells were seeded into 24-well culture plates and cultured for 24 h. The cells were then treated with free drug or nanoparticles for 24 h before being infected with SARS-CoV-2/NTU13 at a multiplicity of infection (MOI) of 5. After 1 h of infection, the virus-containing media was removed, and cells were incubated in RPMI supplemented with 2% FBS and 1% antibiotics. After incubating for 48 h, the culture supernatant was harvested and assayed for the titer of virus as follows. Briefly, a total of 2 × 10^5^ Vero E6 cells were seeded in the 24-well plate one day before being infected at 37 °C with the supernatant containing SARS-CoV-2/NTU13. After 1 h of infection, the cells were rinsed with PBS, covered with a mixture of 1% methylcellulose and DMEM supplemented with 2% FBS, and incubated at 37 °C for 7 days. Cells were then fixed with 10% formaldehyde overnight and stained with 0.5% crystal violet to determine the number of plaques produced. The number of plaques was then used to calculate the virus titer in the supernatant. In the viral yield reduction assay, the amount of SARS-CoV-2 virus RNA was determined by qPCR using the protocol provided by the WHO (https://virologie-ccm.charite.de/en) [41]. RNA from the culture supernatant of Vero E6 cells was extracted and assayed for the E gene using the iTaq Universal Probes One-Step RT-PCR Kit (172–5140, Bio-Rad, USA) and the Applied Biosystems 7500 Real-Time PCR software (version 7500SDS v1.5.1). Plasmid containing partial E fragment was used as the standard to calculate the amount of viral yield (copies/µL). The percentage of inhibition was calculated as 1-(VD/VC), where VD and VC refer to the virus titer in the presence and absence of the test compound, respectively.

#### Western blotting

After the extraction and quantification of the extract protein, 20 µg of protein was subjected to SDS-PAGE as described previously [48]. The target proteins were analyzed by Western blotting protocols using appropriate primary and secondary antibodies. After the primary and secondary antibodies were reacted, the expression of the target protein was analyzed by Western blotting protocols. Antibodies against AXL were purchased from Cell Signaling (Temecula, CA, USA). Antibodies against ACE2 were purchased from Abcam (Cambridge, UK). Antibodies against GAPDH and β-actin were purchased from Santa Cruz Biotechnology (Santa Cruz, CA, USA). Secondary antibodies were purchased from Santa Cruz Biotechnology (Santa Cruz, CA, USA).

### Ponceau S staining

Ponceau S solution was purchased from Sigma-Aldrich. Nanoparticle preparations were stained with 1 mL of Ponceau S solution for 2 min and then washed twice with distilled water to remove background stain by centrifuging at 15,000 × g for 5 min at 4 °C.

#### *In vivo* **biosafety assay**

Six 6-week-old BALB/c male mice (National Laboratory Animal Center, Taipei, Taiwan) were randomly divided into two groups with three mice in each group. The mice in these two groups were treated intratracheally with 50 µL of 0.9% normal saline or 10 µM CM-NP-Q in 0.9% normal saline [49]. After 24 h of treatment, serum and lung were collected for liver function analysis and histological examination, respectively. Liver function was determined by examining the serum levels of aspartate transaminase (AST) and alanine transaminase (ALT) as assayed by an ADVIA2120 hematology system from Siemens Healthineers (Erlangen, Germany). Histological examination of lung was determined by hematoxylin and eosin (H&E) staining of the lung Sect. [48]. All of the mice experiments were approved by the Institutional Animal Care and Use Committee (IACUC) of Chang Gung University (IACUC approval no.: CGU110-015) and Chang Gung Memorial Hospital (IACUC approval no.: 2,020,121,704).

#### Statistical methods

Statistical comparison of multiple-groups results was performed by one-way analysis of variance (ANOVA) with Tukey’s post-hoc by GraphPad Prism Software (Prism Version 5, CA, USA). When *p* < 0.05, it was considered statistically significant.

### Electronic supplementary material

Below is the link to the electronic supplementary material.

Supplementary Material 1

Supplementary Material 2

Supplementary Material 3

Supplementary Material 4

## Data Availability

All data generated for this study are included in the article.

## Declarations

All of the mouse experiments were approved by the Institutional Animal Care and Use Committee (IACUC) of Chang Gung University (IACUC approval no.: CGU110-015) and Chang Gung Memorial Hospital (IACUC approval no.: 2020121704).

## Abbreviations

ACE2: Angiotensin-converting enzyme 2
COVID-19: Coronavirus disease 2019
AXL: AXL tyrosine kinase receptor
NPs: Nanoparticles
NP-Q: NPs encapsulated with quercetin
CM-NPs: ACE2-containing cell membranes nanoparticles
CM-NP-Q: Nanoparticles encapsulated with quercetin and coated with ACE2-membrane
GFP: Green fluorescent protein
MOI: Multiplicity of infection
