# Supplementary figures and images for: Development of nanoparticles incorporated with quercetin and ACE2-membrane as a novel therapy for COVID-19
Source: J Nanobiotechnology. 2024 Apr 12;22:169. doi: 10.1186/s12951-024-02435-2 (PMC11015574; doi:10.1186/s12951-024-02435-2)

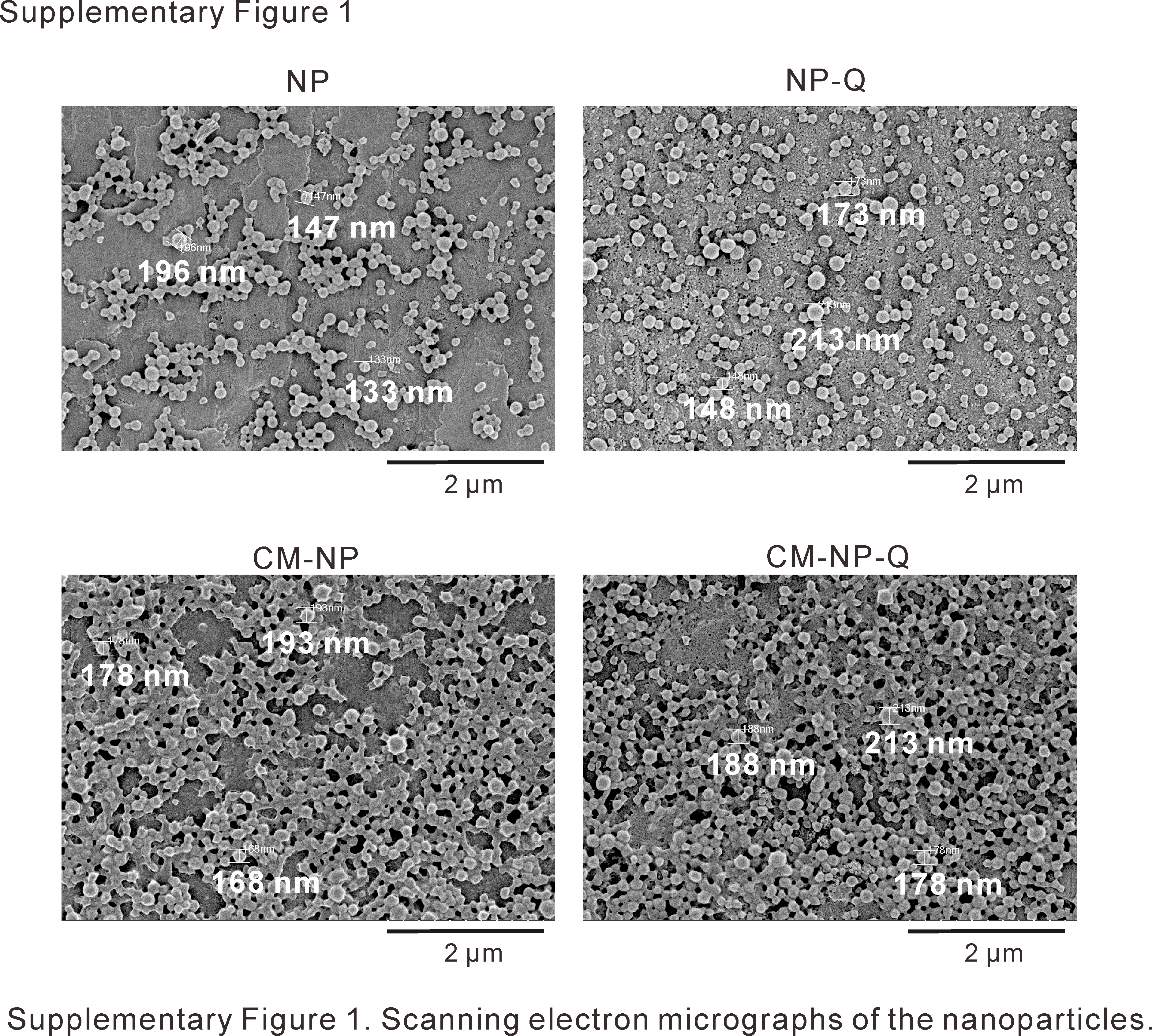

Supplement: Supplementary file 1 — Supplementary Material 1 [file 12951_2024_2435_MOESM1_ESM.tif]
