# Supplementary material for: Development of nanoparticles incorporated with quercetin and ACE2-membrane as a novel therapy for COVID-19
Source: J Nanobiotechnology. 2024 Apr 12;22:169. doi: 10.1186/s12951-024-02435-2 (PMC11015574; doi:10.1186/s12951-024-02435-2)
Supplement: Supplementary file 3 — Supplementary Material 3 [file 12951_2024_2435_MOESM3_ESM.docx]

Supplementary Table 1. The particle size, zeta potential, polydispersity index and entrapment efficiency of nanoparticles encapsulated with quercetin and/or coated ACE2-containing cell membranes^a^

| Formulation | size (nm) | zeta potential (mV) | polydispersity index (PDI) | entrapment efficiency (%) |
| --- | --- | --- | --- | --- |
| NP | 177.1 ± 2.19 | -4.45 ± 1.28 | 0.113 ± 0.013 |  |
| NP-Q | 181.6 ± 5.55 | -13.34 ± 2.18 | 0.160 ± 0.026 | 98.53%± 0.37 |
| CM-NP | 202.3 ± 14.36 | -13.83 ± 1.09 | 0.206 ± 0.08 |  |
| CM-NP-Q | 218.6 ± 29.40 | -14.61 ± 2.33 | 0.248 ± 0.055 |  |

^a^Data are expressed as mean ± SD (n=3).

The true PDI is Z average only.
